# The Validity of Nursery Teachers’ Report on the Physical Activity of Young Children

**DOI:** 10.2188/jea.12.367

**Published:** 2007-11-30

**Authors:** Xiaoli Chen, Michikazu Sekine, Shimako Hamanishi, Hongbing Wang, Yasuko Hayashikawa, Takashi Yamagami, Sadanobu Kagamimori

**Affiliations:** 1 Department of Welfare Promotion and Epidemiology, Toyama Medical and Pharmaceutical University, Faculty of Medicine; 2 Hokuriku Health Service Association

**Keywords:** preschool children, physical activity, questionnaire, validity, observational evaluation

## Abstract

This study examined the validity of nursery teachers’ report on the physical activity of young children. Subjects were twenty-one children aged 3 to 4 years (12 boys and 9 girls) at a nursery in Toyama Prefecture, Japan. Children were equipped with the Actiwatch (Mini-mitter Company Inc.) activity monitor and the Caloriecounter Select II (Kenz, Co, Ltd) for three consecutive weekdays to assess their daily physical activity levels. Nursery teachers completed a questionnaire containing questions on children’s activity level during the measurement periods at the nursery. The results showed that subjects with a high frequency of physical activity were significantly associated with an increasing trend in total energy expenditure and activity counts per day. Children whose physical activity was rated as “very often” had a significantly higher activity level per day from the Actiwatch instrument, compared with peers whose physical activity was rated as “not often” (570.5±192.8 counts vs. 334.9±123.4 counts, p<0.05). Regarding energy expenditure originating from physical activity and steps per day from the Caloriecounter, a significant difference was found between “very active” children and “inactive” children as rated by the nursery teachers (140.7±17.5 kcal vs. 78.2±17.4 kcal, p<0.05; 16103±1896 steps vs. 10038±32 steps, p<0.05). This study indicates that children’s physical activity level as reported by their teachers in nursery surroundings is in accordance with the objective data from the Actiwatch and the Caloriecounter. The results suggest that nursery teachers’ respondent for children in physical activity may be used as a valid measure to evaluate young children’s physical activity levels, especially in nursery setting.

A growing body of literature suggests that adequate amounts of physical activity can improve cardiovascular fitness, build muscular strength and endurance, and decrease the risk of obesity and several hypokinetic diseases, and are associated with improvements in mental health and health-related quality of life.^1^^,^^2^^,^^3^ Substantial attention has been paid to the activity levels of children and adolescents, largely because of changing lifestyles that have reduced the opportunity for physical activity and also introduced attractive sedentary alternative such as the playing of computer games.^4^

Assessment of physical activity in preschool children has been undertaken to describe the present levels of physical activity in these individuals, to define a setting (day-care, playground), and to evaluate interventions that are used to increase the level of physical activity.^5^ For young children, the greatest portion of accumulated physical activity perhaps comes from lifestyle activities which include active play and games involving the large muscles of the body.^4^

In epidemiologic studies, some kinds of structured questionnaires have been used for children and adults.^6^^,^^7^ However, there is not much research work on young children’s engagement in the reporting of the level of physical activity. In young children such as in a nursery setting or kindergarten environment, it is not yet clear whether teachers’ reports about young children’s physical activity is suitable for a large scale epidemiologic study.

The purpose of this study was to examine the validity of nursery teachers’ report on the physical activity of young children. It was performed at a nursery in Toyama to make a confirmation whether nursery teachers’ subjective reports on physical activity levels of young children could be validated by using two small instruments, namely the Actiwatch and the Caloriecounter, as objective measures.

## METHODS

### Subjects

This survey was conducted at a town’s nursery in July and August 2001, as a sub-study of the Toyama Birth Cohort Study in Toyama Prefecture, Japan. Out of a total of 55 children aged 3 to 4 years at the nursery, parental permission to participate in the study was obtained from 24 healthy children (14 boys and 10 girls). Written consent was obtained from the children’s parents. All the children were free of any chronic or current health problems. Of the 24 subjects, one child was reluctant to continue the survey and thus the observation on this child stopped. Data from two other children were unavailable because of measurement failure from the instruments. Therefore, the present study subjects consist of 21 children (12 boys and 9 girls).

### Actiwatch and Accelemetor:

Actiwatch-L (Mini Mitter Company, Inc., Bend, OR) is a small, lightweight (17 grams), limb-worn, activity-monitoring watch-like computerized device. It has been used in sleep studies^8^^,^^9^ and physical activity,^4^ and is beginning to be used in energy expenditure studies.^10^ The Actiwatch activity monitor contains an omni-directional sensor capable of detecting acceleration in two planes. Sensitive to 0.01 gravity (0.098m/s^2^), this type of sensor integrates the degree and speed of motion and produces an electrical current that varies in magnitude. An increased degree of speed and motion produces an increase in voltage. The monitor stores this information as activity counts. This device measures long-term gross motor activity and ambient light exposure.^11^ The activity counts are calculated based on the sampling epoch and the total number of activity counts is compared to the threshold sensitivity value selected by the researcher. In this study, a 1-minute sampling epoch was used and the threshold sensitivity “Auto” was selected. Mean activity is the average activity counts during the 24 hours; Diurnal activity is the magnitude of activity each day during the diurnal time period from 5 am through 7 pm; Nocturnal activity is the magnitude of activity each day during nighttime from 7 pm through 5 am.

The Caloriecounter (Calorie Counter Select II, Kenz, Japan) is a small instrument that can calculate children’s daily steps, energy expenditure originating from physical activity, and total energy expenditure per day, as well as the subject’s basal metabolic rate and the minimum of physical activity. This instrument equips a small counter measuring amplitude and frequency of acceleration of body movement every 4 seconds and grades the intensity as one of 10 levels. The graded intensity is transformed into the corresponding coefficient of physical activity (Ka; coefficient as determined by the level of physical activity (kcal/kg/4 seconds). The energy expenditure originating from physical activity per 4 seconds(C) is calculated as Ka×W (weight in kg). The basal metabolism (B) is also calculated as follows: B=Kb×A×T

(Kb: standard value for basal metabolism per body surface area (kcal/m^2^/hr); A: body surface area (cm^2^)=W^0.444^×H^0.663^ (height in cm)×88.83; T: time in hour). Energy expenditure originating from minimum body movement (X(kcal/4sec)) is calculated every 4 seconds as follows: X=Kx×B

(Kx: coefficient for minimum body movement as estimated by energy metabolic rate; B: basal metabolism)

Finally the total energy expenditure (E) is calculated as follows: E= 10/9(B+C+X).

The total energy expenditure and energy expenditure originating from physical activity are calculated every 4 seconds and summed during 24 hours (TEE: total energy expenditure per day; EEPA: energy expenditure originating from physical activity per day). Steps per day are automatically counted by the Caloriecounter instrument.

Detailed information of the methodology for calculating the indices obtained from this small instrument and the validity of these measurements have been published elsewhere.^6^^,^^12^

### Procedures

Anthropometric measurements were conducted at the nursery. The heights and weights of children were measured twice in their shorts by one trained examiner. Height was measured to the nearest 0.1 centimeter by using a rigid stadiometer, while weight was measured to the nearest 0.1 kilogram by using a weighing scale. Then investigators attached the monitoring instruments: the Actiwatch to children’s non-dominant ankle and the Caloriecounter to the waist by means of a belt-worn for nocturnal and daytime activity monitoring except during the bathing/shower time. Children were equipped with the Actiwatch activity monitor and the Caloriecounter Select II for three consecutive weekdays, such as from Monday morning to Thursday morning. The two monitoring instruments were also detached at the nursery by the investigators.

On the other hand, three nursery teachers who were in charge of their classes completed a questionnaire about children’s physical activity level at the nursery during the past one week. Since children from three classes participated in the survey, the three nursery teachers who took charge of their classes individually evaluated the children’s physical activity levels in their classes. The questionnaire included: the child’s preference for physical activity, rated on a three-point scale (1 “like very much” to 3 “do not like”); the frequency of taking part in exercise, rated on a three-point scale (1 “very often” to 3 “not often”) and the level of activity, rated on a 4-point scale (1 “very active” to 4 “inactive”). All the rating scales were based on a comparison to their peers in the class during the past one week. The questions and the rating scales in the questionnaire were based on the Toyama Cohort Study (the 1st and 2nd Survey), when children aged 3 and 6 years old, respectively.^13^^,^^14^

### Statistical analysis

Gender difference in activity parameters was evaluated by two-sample t test. A test for linear trend in one-way analysis of variance (ANOVA) was performed to evaluate the significance of the linear trend of the mean of the objective indices among groups with different physical activity level as reported by nursery teachers. We further used Bonferroni test of multiple comparison to assess the difference between different physical activity levels, if p value was less than 0.05 by ANOVA.

All statistical analyses were performed by the SPSS 7.5.1J software package. A two-tailed p value of less than 0.05 was considered to be significant.

## RESULTS

Representative Actiwatch actograms obtained from two children who were rated as “very active” or “inactive” by the nursery teachers are shown in Figure. The boy rated as “very active” by his nursery teacher had a mean activity of 547 counts per day and highest activity count of 6069, whereas the mean activity of the “inactive” 4 years old boy was 121 counts per day with a highest count of 3589 during the measurement periods. On the other hand, the mean activity of a 4-year-old girl rated as “ordinary” was 371 counts per day with a highest activity count of 5201 counts during the same observation period.

**Figure.**
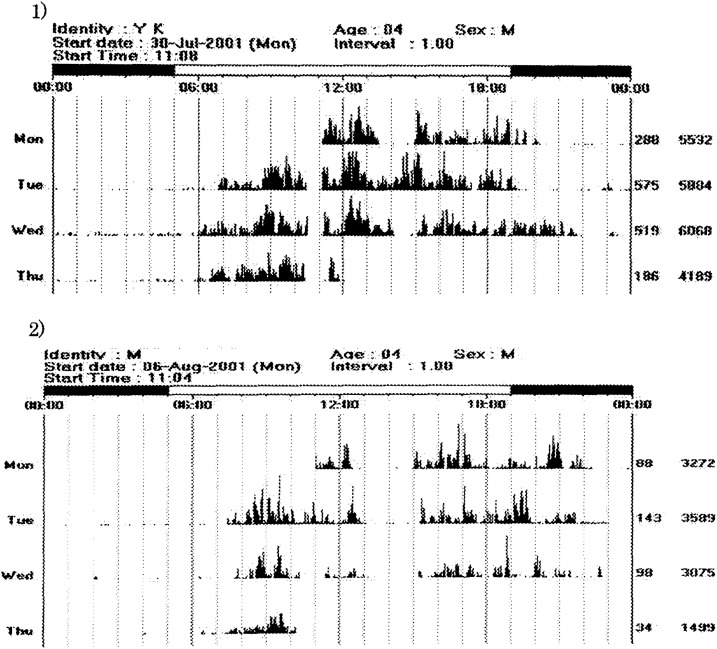
Actigraphic data for three typical successive nights for a “ very active” boy aged 4 and an “inactive” boy as rated by their nursery teachers. 1) Actigraphic data of a “very active” boy aged 4. The mean activity over three days for the “very active” child was 547 counts. The highest number of activity counts for a single sampling epoch was 6069 during the study period. 2) Actigraphic data of an “ inactive” boy aged 4. The mean activity over three days for the “inactive” child was 121 counts. The highest number of activity counts for a single sampling epoch was 3589 during the study period. Activity: leg activity data compressed to one-minute epoch, diurnal time, 5 am through 7 pm; nocturnal time, 7 pm through 5 am.

The general characteristics of the study subjects are presented in Table 1. The mean age of the study subject was 3.8 years old (standard deviation:0.26) and the mean weight and height were 15.5 ± 1.25kg (mean ± standard deviation) and 98.8 ± 3.01cm, respectively. The mean BMI was 15.8kg/m^2^ (boys:15.6 ± 0.69kg/m^2^, and girls:16.1 ± 0.90kg/m^2^). Age, height, weight, and BMI showed no significant difference between boys and girls. The descriptive statistics for the activity parameters are as follows: total energy expenditure per day (TEE) was 1161.9 ± 66.9 kcal (boys:1192.9±66.2 kcal, girls:1120.6±42.7 kcal, respectively), with the boys’ expenditure being significantly higher than girls’ (p<0.05). Otherwise, the level of children’s activity was not significantly different between the two genders.

**Table 1.** The descriptive characteristics of 21 nursery children aged 3-4 years old

variable	total		boys		girls		p value
	
	(n=21)		(n=12)		(n=9)		
	Mean	SD	Mean	SD	Mean	SD	
age (year)	3.8	0.26	3.8	0.24	3.9	0.29	0.978
height (cm)	98.8	3.01	99.5	2.98	97.9	2.97	0.230
weight (kg)	15.5	1.25	15.5	1.18	15.5	1.40	0.977
BMI (kg/m^2^)	15.8	0.80	15.6	0.69	16.1	0.90	0.172
data from the Caloriecounter							

BEE (kcal)	793.4	34.17	814.9	28.76	764.8	12.85	0.001
TEE (kcal)	1161.9	66.92	1192.9	66.16	1120.6	42.65	0.010
EEPA (kcal)	110.1	29.56	107.4	29.75	113.6	30.70	0.646
steps per day (steps)	12638	2849	12416	3298	12934	2273	0.691
data from the Actiwatch							
mean activity (counts)	404.5	155.08	447.6	167.14	347.1	123.41	0.118
diurnal activity (counts)	503.5	201.31	556.7	214.50	432.6	167.81	0.168
nocturnal activity (counts)	175.1	106.72	209.4	117.46	129.2	73.24	0.088

Abbreviations:

BMI, body mass index; BEE, basal metabolism; TEE, total energy expenditure per day; EEPA, energy expenditure originating from physical activity per day

Table 2 shows the results of the trend test and Bonferroni test of the relation between the objective indices from the two monitoring instruments and the frequency of children’s physical activity. Subjects with a high frequency of physical activity were significantly associated with an increasing trend in total energy expenditure and activity counts per day. Children whose physical activity was rated as “very often” had a significant higher level of energy expenditure per day from the Caloriecounter and higher activity counts per day from the Actiwatch, when compared with peers whose physical activity was rated as “not often” (1257.1 ± 101.9 kcal vs. 1148.1 ± 71,4 kcal, p<0.05; 570.5 ± 192.8 counts vs. 334.9 ± 123.4 counts, p<0.05; respectively). There were no significant differences in the children’s age, weight, height, and BMI among the three groups.

**Table 2.** The frequency of children’s physical activity as evaluated by nursery teachers

variable	very often		fairly often		not often		ANOVA

	(n=3)		(n=13)		(n=5)		

	Mean	SD	Mean	SD	Mean	SD	p value
age (year)	3.69	0.15	3.89	0.27	3.68	0.26	0.694
height (cm)	99.23	4.40	98.08	2.43	98.94	4.88	0.984
weight (kg)	15.50	1.80	15.29	0.93	15.46	1.99	0.999
BMI (kg/m^2^)	15.70	0.72	15.90	0.76	15.73	1.01	0.885
TEE (kcal)	1257.1	101.9	1156.2	57.43	1148.1	71.4	0.019
EEPA (kcal)	140.6	17.5	99.6	30.7	105.88	28.1	0.154
steps per day (steps)	16103	1896	11572	2808	12368	2193	0.064
mean activity (counts)	570.5	192.8	428.1	385.2	334.9	123.4	0.026
diurnal activity (counts)	796.1	194.9	569.0	495.8	486.7	191.7	0.014
nocturnal activity (counts)	278.7	211.3	230.8	234.8	122.4	66.2	0.129

Abbreviations: BMI, body mass index; TEE, total energy expenditure per day; EEPA, energy expenditure originating from physical activity per day

Note: multiple comparison (Bonferroni test)

TEE: * p<0.05 very often>fairly often; very often>not often

mean activity: * p<0.05 very often>fairly often; very often>not often

diurnal activity: * p<0.05 very often>fairly often; very often>not often

The mean levels of indices among groups with a different preference in physical activity are presented in Table 3. When compared to peers whose physical activity was rated as “like”, those who were rated as “like very much” had higher energy expenditure from physical activity, steps per day and diurnal activity counts, although significant differences were not found. Only one child in this study was rated as “do not like” physical activity, who had a low activity level of 333.4 counts and a total energy expenditure of 1038.6 kcal per day. There were significant difference in children’s mean activity counts per day, those who were rated as “like very much” had a higher activity counts than those whose physical activity were rated as “like”(p<0.05).

**Table 3.** Different preferences for physical activity as evaluated by nursery teachers.

variable	like very much		like		p value

	(n=5)		(n=15)		

	Mean	SD	Mean	SD	
age (year)	3.75	0.15	3.90	0.26	0.249
height (cm)	99.34	3.27	99.12	2.36	0.871
weight (kg)	15.80	1.35	15.60	0.89	0.706
BMI (kg/m^2^)	15.99	0.66	15.88	0.81	0.793
TEE (kcal)	1214.4	106.9	1152.6	31.1	0.052
EEPA (kcal)	126.3	23.6	107.5	29.3	0.213
steps per day (steps)	14211	2924	12286	2769	0.200
mean activity (counts)	535.5	148.6	365.6	141.1	0.033
diurnal activity (counts)	639.6	268.1	449.4	162.4	0.070
nocturnal activity(counts)	238.9	172.6	161.3	71.8	0.162

Abbreviations: BMI, body mass index; TEE, total energy expenditure per day; EEPA, energy expenditure originating from physical activity per day

Table 4 provides a comparison of children evaluated as very active, active, ordinary or inactive by the nursery teachers and the level of measured activity. There were significant increasing trends in the relation between several objective indices and the level of physical activity levels rated by teachers. Children who were rated as “very active” had significant higher levels in total energy expenditure per day, energy expenditure originating from physical activity, daily steps from the Caloriecounter and mean activity from the Actiwatch, when compared to peers who were inactive at the nursery (p<0.05). No significant differences in the children’s age, weight, height, and BMI among the four groups were found.

**Table 4.** Young children’s physical activity levels as evaluated by nursery teachers

variable	very active		active		ordinary		inactive		ANOVA

	(n=3)		(n=5)		(n=11)		(n=2)		
	Mean SD		Mean SD		Mean SD		Mean SD		p value
age (year)	3.69	0.15	3.97	0.20	3.89	0.25	3.43	0.10	0.501
height (cm)	99.23	4.40	99.34	1.61	98.89	2.58	96.25	7.14	0.403
weight (kg)	15.50	1.80	15.80	0.45	15.50	0.94	14.50	3.54	0.473
BMI (kg/m^2^)	15.70	0.72	16.02	0.56	15.80	0.89	15.50	1.51	0.821
TEE (kcal)	1257.1	101.9	1145.5	57.0	1149.8	27.6	1126.7	124.4	0.024
EEPA (kcal)	140.6	17.5	110.3	34.7	107.5	26.2	78.2	14.7	0.030
steps per day (steps)	16103	1896	12105	2978	12409	2611	10038	32.0	0.031
mean activity (counts)	570.5	192.8	399.7	65.7	378.4	138.4	215.2	165.8	0.002
diurnal activity (counts)	796.1	194.9	453.8	110.3	465.8	162.8	396.6	336.2	0.020
nocturnal activity (counts)	278.7	211.3	161.53	75.57	172.8	76.42	65.5	6.94	0.064

Abbreviations: BMI, body mass index; TEE, total energy expenditure per day; EEPA, energy expenditure originating from physical activity per day

Note: multiple comparison (Bonferroni test)

TEE: * p<0.05 very active>active; very active >inactive

EEPA: * p<0.05 very active>ordinary; very active >inactive

steps per day: * p<0.05 very active>active; very active >inactive

mean activity: * p<0.05 very active>active; active>ordinary; very active >inactive

diurnal activity: * p<0.05 very active>active; very active>ordinary; very active >inactive

## DISCUSSION

Most researchers agree that providing children with opportunities to increase physical activity levels and enhance health-related fitness is important and school physical education is recognized as the most widely available resource for promoting physical activity among children and adolescents.^15^^,^^16^

Accurate assessment of physical activity in children is necessary to identify current levels of activity and to assess the effectiveness of intervention programmes designed to increase physical activity.^17^ Physical activity can be assessed by subjective and objective methods that include questionnaire, direct observation, and mechanical devices.^18^^,^^19^ The physical activity questionnaire is probably the most commonly used method to assess physical activity in population studies because of its relatively low cost and ease of administration.^20^^,^^21^ Self-reported questionnaires evaluating the physical activity level of adolescents and adults have been widely used in epidemiologic survey.^22^^,^^23^ These techniques must be used cautiously in a paediatric population that has difficulty recalling such information.^17^ In a review of children’s and adolescents’ physical activity patterns, Fox and his co-workers concluded that the self report of activity was unreliable with young children, and objective measures are required that are cheap and effective for large samples.^4^

Objective measures of physical activity are often included in a study to validate the data from the questionnaires. It has been previously validated as an objective monitor of children’s physical activity in field and laboratory settings.^17^ Data indicate that in field settings, accelerometry can be used to assess the intensity of children’s activity.^7^^,^^18^ The widely used method to validate the assessment of physical activity is the use of an activity monitor such as Caltrac, Tritrac, or the CSA accelerometer.^4^^,^^5^^,^^7^

This study examined the relationship between young children’s activity levels as evaluated by the Actiwatch and the Caloriecounter, and nursery teachers’ subjective evaluation about children’s activity levels at a nursery. The results indicate that the reports from nursery teachers are in accordance with the objective data from the Actiwatch or the Caloriecounter. No sex difference was found in the activity parameter in the present study, which is consistent with other studies.^24^^,^^25^ Fairweather^26^ reported a relatively high correlation between direct observation and the CSA accelerometer during a preschool exercise results. Using whole room calorimetry as a criterion measure of energy expenditure, Treuth^27^ assessed the validity of simultaneously measuring heart rate and leg accelerometry to estimate energy expenditure of 20 children aged 8-12 years old. Noland^28^ observed little correlation between direct observation and either a teacher’s or a parent’s rating of the child’s activity. Two other studies observed significant associations using either a teacher report^29^ or a parent report.^30^

Although it is tempting to think that parents would provide an accurate assessment of their child’s activity, this does not always seem to be the case.^29^ In the nursery or kindergarten setting, as well as in the school environment, parental reports may have certain strengths (for example, parents’ observation of children’s activity patterns in their home) and some limitations in their capacity to document children’s physical activity levels, such as parents’ limited knowledge about their children’s daily activity levels during weekdays in a nursery or school setting.

In this study, physical activity counts was significantly related to the data on TEE (partial relation coefficient r=0.51, p<0.05) and EEPA (partial relation coefficient r=0.72, p<0.01), after adjusting for age, gender, weight, and height. Then, the positive relationship between physical activity and TEE may not be explained by the difference in age, sex, height, and weight among the different groups evaluated by teachers.

Some limitations may be identified with regard to the interpretation of data from this study. Firstly, the study was conducted between July and August in 2001. Children at the nursery played with water in a small pool for thirty minutes every day during the study period. The other limitation is the small number of children included in the study. Lastly, teachers’ report for young children’s physical activity could be easy to understand to introduce additional sources of bias, such as observation bias. However, during the measurement periods, the 21 children took their bath under the same conditions and the children’s shower time was about 10 minutes. Therefore, children’s behaviors relating to taking off the two apparatus should not have a great effect on the actual data collection originating from the Actiwatch or the Caloriecounter. On the other hand, the levels of children’s physical activity as rated by nursery teachers were strongly associated with the levels of the objective indices from the Actiwatch and the Caloriecounter. Thus the results may not be distorted by an increase in the number of subjects. Researchers explained the intention of this study to the principal of the nursery before the survey. And the nursery teachers who took charge in their classes completed the questionnaire without receiving such the detailed explains as the principal of the nursery got. Teachers evaluated children’s physical activity level in their classes during the past one week, compared with their peers. Although observational bias is inevitable, the observation bias or interviewer bias may not be considered to affect the results to a great degree.

In conclusion, this study indicates that children’s physical activity level as reported by their teachers in nursery surroundings is in accordance with the objective data from the Actiwatch and the Caloriecounter. Although there is limited information for teachers’ reports about physical activity measure in young children, the findings suggest that nursery teachers’ respondent for children in physical activity may be used as a valid measure to evaluate young children’s physical activity levels, especially in nursery setting.
